# Endoscopic-assisted percutaneous fixation for displaced anterior inferior iliac spine avulsion fractures: a prospective cohort study

**DOI:** 10.1186/s10195-025-00831-4

**Published:** 2025-03-08

**Authors:** Andrea Audisio, Alessandro Aprato, Virginia Reinaudo, Giuseppe Sinatra, Lorenzo Lucchino, Alessandro Massè

**Affiliations:** 1 Pediatric Orthopaedics and Traumatology, Regina Margherita Children’s Hospital, Piazza Polonia 94, 10126 Turin, Italy; 2 Department of Surgical Sciences, University of Turin, Turin, Italy; 3 Trauma and Orthopaedic Centre, Città Della Salute e Della Scienza, Turin, Italy

**Keywords:** Anterior Inferior Iliac Spine, Avulsion Fracture, Endoscopic-Assisted Fixation, Subspine Impingement, Pediatric Sports Injuries, Prospective Cohort Study

## Abstract

**Introduction:**

Anterior inferior iliac spine (AIIS) avulsion fractures commonly occur in adolescent patients during sports activities. To systematically evaluate fracture severity and guide management, an adaptation of the Hetsroni classification system was used to categorize fractures on the basis of their displacement relative to the acetabular rim. Traditional open reduction and internal fixation reported satisfactory consolidation rates but complications such as lateral femoral cutaneous nerve (LFCN) neuropathies, heterotopic ossifications (HO), and subspine impingement. The objectives of this work are to (1) report short- and mid-term radiographic and clinical outcomes and (2) propose an adapted classification system based on the risk of subsequent subspine impingement.

**Materials and methods:**

A prospective cohort study was conducted on patients with AIIS avulsion fracture with ≥ 1.5 cm displacement who underwent surgery between 2021 and 2024. Patients with follow-up < 6 months, displacement < 1.5 cm, comminuted fractures, or chronic fractures were excluded. Clinical outcomes, including the subspine impingement test, the modified Harris Hip Score (mHHS), and the University of California Los Angeles Score (UCLA), were evaluated at last follow-up. Postoperative complications, such as LFCN neurapraxia, HO (classified by Brooker), and surgical revisions, are reported.

**Results:**

Eleven male patients with mean age of 14.1 years (range 12.8–15.0 years) were included. Fractures were classified as type I in two patients (18.2%), type II in four patients (36.4%), and type III in five patients (45.4%). The mean surgical duration was 71.4 min (SD 17.1 min), and the average time from injury to surgery was 4.2 days (range 1–11 days). The mean fracture displacement was 18.3 mm (range 15–25 mm). Postoperative scores averaged 89.7 for mHHS (SD 3.1) and 9.7 for UCLA (SD 0.6). Patients were followed for 20.0 months (range 6–47 months, SD 13.3 months). One patient underwent open surgical revision and subsequently experienced temporary LFCN neurapraxia, HO (Brooker 1), and symptoms of subspine impingement.

**Conclusions:**

Endoscopic-assisted percutaneous fixation is an effective technique for treating displaced AIIS avulsion fractures. Preliminary results suggest that this approach offers noninferior results, satisfactory outcomes, and limited complications. Further studies with long-term follow-up are needed to confirm these findings.

## Introduction

Avulsion fractures of the anterior inferior iliac spine (AIIS) are relatively common pelvic injuries, accounting for approximately one-third of pelvic avulsions in adolescents [1–3]. These fractures primarily occur in male teenagers owing to abrupt and intense contractions of the rectus femoris muscle or eccentric muscle lengthening during activities, coinciding with the open growth plate [4, 5]. Hormonal changes during adolescence contribute to muscle strengthening, which, coupled with the ongoing secondary ossification of the apophyses, increases the susceptibility to these injuries [4]. Anteroposterior and alar-view pelvis X-rays are effective as primary imaging tools for diagnosing AIIS avulsion fractures [6]. However, in some cases, advanced three-dimensional imaging techniques such as computed tomography (CT) and magnetic resonance imaging (MRI) may be necessary for a more precise assessment of fragment position and displacement, to select the most appropriate treatment strategy [5, 7].

The management of AIIS avulsion fractures remains a subject of ongoing debate [7]. Traditionally, these fractures have been treated conservatively, with the understanding that most cases heal successfully without surgical intervention. Studies have demonstrated excellent outcomes with nonoperative management, even in cases with radiographic displacement [8–13]. However, these findings are often limited by short follow-up periods, leaving the long-term functional and radiological outcomes less well defined.

In recent years, increasing attention has been paid to the surgical management of AIIS avulsion fractures, particularly for preventing secondary complications [1, 4]. Subspine impingement, a form of extraarticular femoroacetabular impingement (FAI), has been identified as a common sequela of inadequately managed or displaced fractures [14]. This condition, characterized by abnormal contact between the femur and the AIIS region, can result in pain and restricted hip mobility [14]. Both open [15–19] and arthroscopic techniques [14, 20–22] have been described to address this issue, with promising results in terms of alleviating symptoms and restoring function. Despite these advancements, a clear consensus on the indications for surgical intervention in acute AIIS avulsion fractures remains elusive [7].

To standardize the assessment of fracture displacement and guide clinical decision-making, we adapted the Hetsroni classification system [23], originally developed to describe AIIS morphology in femoroacetabular impingement. This classification categorizes AIIS variants on the basis of their relationship to the acetabular rim and associated biomechanical implications. In our study, acute AIIS fractures were classified into three types: type I (displacement proximal to the acetabular rim), type II (displacement aligned with the acetabular rim), and type III (displacement extending distal to the acetabular rim). This adaptation may improve the assessment of fracture severity and risk of subspine impingement by analyzing the relationship between AIIS displacement with respect to the acetabular rim [23, 24].

This study evaluates the outcomes of endoscopic-assisted reduction and internal fixation (EARIF) for displaced AIIS avulsion fractures [25]. The objectives are to (1) assess short- and mid-term radiographic and clinical outcomes and (2) introduce an adapted classification system based on the risk of subspine impingement.

## Materials and methods

### Fracture classification

**Fig. 1:**
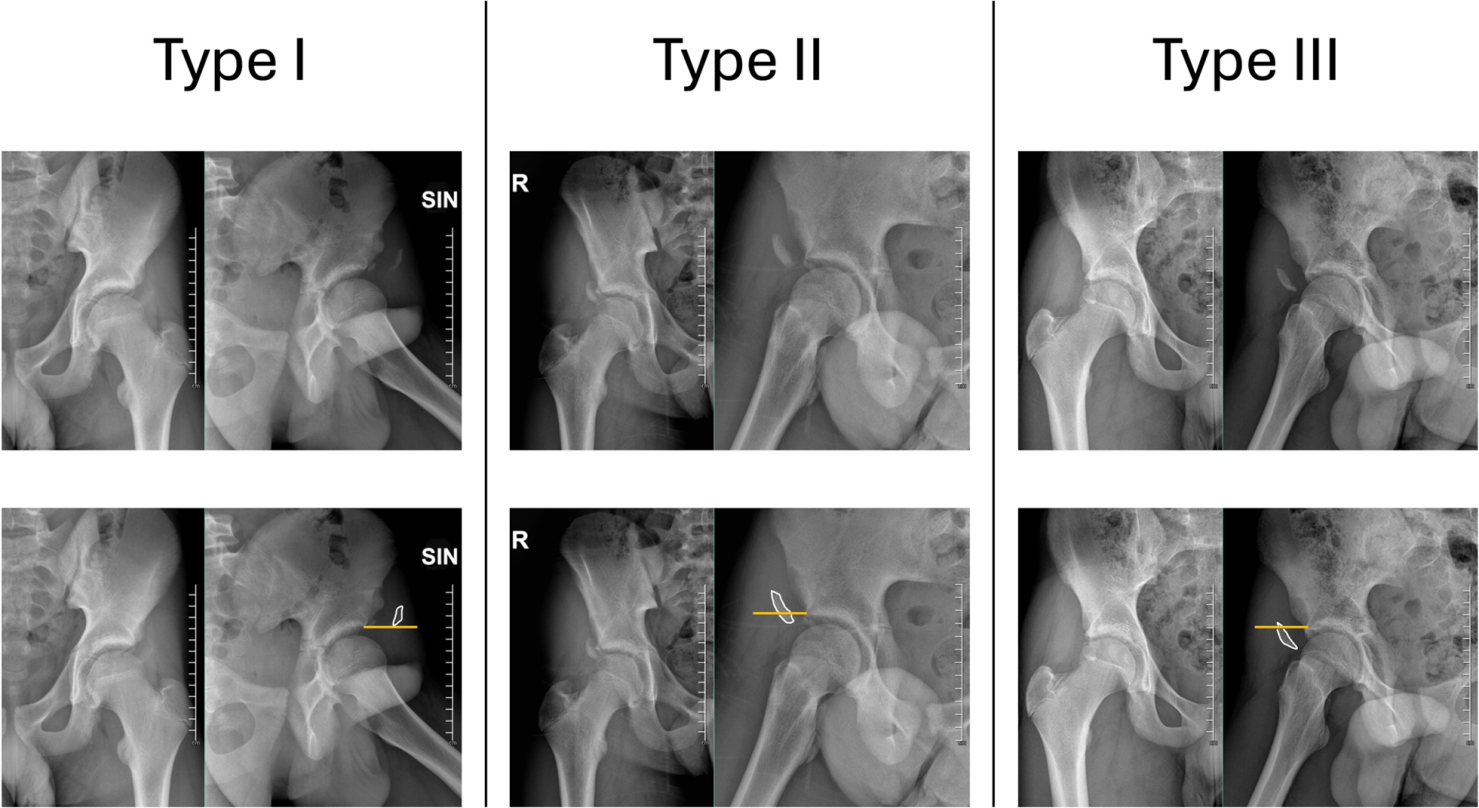
Adapted classification system for anterior inferior iliac spine (AIIS) avulsion fractures, based on the Hetsroni et al. classification [23]. This system categorizes fractures into three types according to the displacement of the fracture fragment relative to the acetabular rim and the associated risk of subspine impingement: type I, fragment’s displacement located proximal to the acetabular rim; type II, fragment’s displacement aligned with the acetabular rim; and type III, fragment’s displacement extending distal to the acetabular rim

To systematically evaluate the severity of fracture displacement and its potential impact on outcomes, we adapted the Hetsroni classification system [23], originally developed to describe AIIS morphology in femoroacetabular impingement. Acute fractures were categorized into three types on the basis of their relationship to the acetabular rim (Fig. 1):

Type I: displacement proximal to the acetabular rim

Type II: displacement aligned with the acetabular rim

Type III: displacement extending distal to the acetabular rim

Radiographic classification was performed using standardized anteroposterior (AP) and alar iliac views of the pelvis according to Judet [26], assessed by a senior surgeon who was not directly involved in patient care to ensure objectivity.

### Study design

This retrospective analysis of prospectively collected data included a consecutive series of patients with anterior inferior iliac spine (AIIS) avulsion fractures between January 2021 and May 2024. Inclusion criteria for AIIS endoscopic-assisted reduction and internal fixation (EARIF) was displacement ≥ 1.5 cm [25] or if classified as type II or III according to the modified Hetsroni classification. Surgical indication was based on the fact that type II and III fractures were associated with a reduction of hip flexion and internal rotation without any mention to the amount of displacement [7, 22, 23].

Exclusion criteria were displacement of < 1.5 cm and classified as type 1 according to the modified Hetsroni classification, which were treated nonoperatively [7, 27]. Moreover, patients with follow-up < 6 months, comminuted fractures, and chronic fractures were not included in the study.

All procedures were performed at a large teaching referral center by one senior surgeon experienced in hip arthroscopy and pediatric trauma (A.Ap.). Informed consent was obtained from all patients and their legal guardians. The study protocol received a waiver from the local ethics committee.

### Interventions

#### Outcomes

Demographic data were collected, including gender, age at the time of surgical treatment, and the time interval from symptom onset to surgery.

Radiographic evaluations were performed by a senior surgeon not directly involved in patient care to ensure objectivity. Preoperative fracture displacement was assessed using anteroposterior (AP) and Judet views of the pelvis. Intraoperative parameters, including surgical duration, were recorded.

Clinical outcomes were measured at the final follow-up and included the subspine impingement test, the modified Harris Hip Score (mHHS) [28], and the University of California Los Angeles (UCLA) activity score [29, 30]. Postoperative complications, such as lateral femoral cutaneous nerve (LFCN) neurapraxia, heterotopic ossifications (HO, classified by Brooker) [31], and the need for surgical revisions, were systematically documented.

#### Surgical technique

**Fig. 2:**
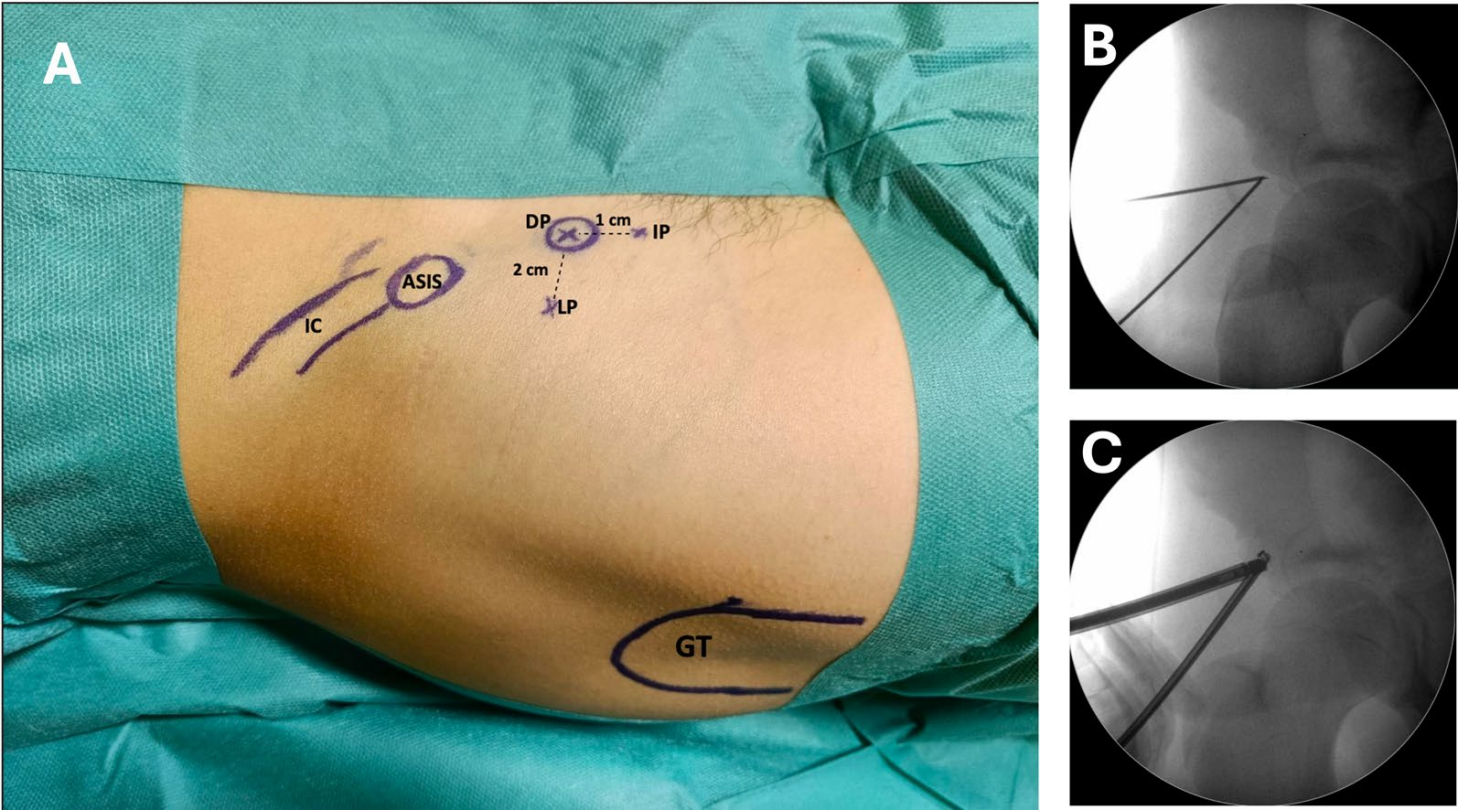
**A** Endoscopic portals are placed using the ASIS bony landmark and AIIS avulsed fragment using fluoroscopic assistance: IC: iliac crest, ASIS: anterior superior iliac spine, GT: greater trochanter, DP: direct portal, directly on the anterior inferior iliac spine, IP: inferior portal, 1 cm inferiorly to AIIS, LP: lateral portal, 2 cm lateral to AIIS. **B** Fluoroscopic view of Nitinol wires placed on the fracture site. **C** Camera is inserted through the LP while the readiofreuency is inserted through the DP

An updated version of the EARIF technique [25] was performed in all cases. The patient is placed on a radiolucent operating table, with the affected limb left free to allow hip flexion during surgery if needed. Using intraoperative fluoroscopy, the anterior inferior iliac spine (AIIS) is identified, and three endoscopic portals are established: the lateral portal (LP), direct portal (DP), and inferior portal (IP) (Fig. 2A).

The procedure begins with the insertion of Nitinol wires through the LP and DP portals (Fig. 2B). A 30° arthroscope is introduced via the DP, and radiofrequency is used via the LP (Fig. 2C). Water pressure is set at 40 mmHg. Care must be taken when approaching the LP to avoid excessive lateral placement, which risks encroaching on the lateral femoral cutaneous nerve (LFCN). On the medial side, careful attention is needed to avoid femoral vascular and nerve structures when placing the DP and IP portals.

**Fig. 3:**
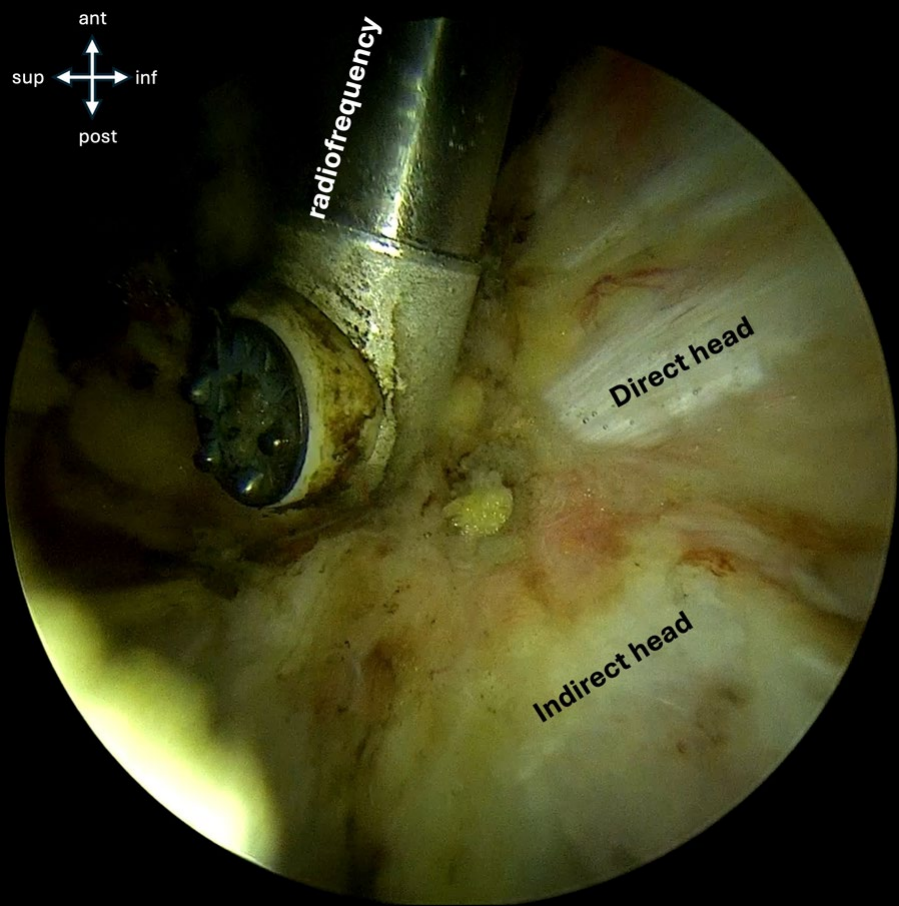
Endoscopic view shows the direct and indirect heads of the rectues femoris, the radiofrequency probe, and superiorly, the fracture site

**Fig. 4:**
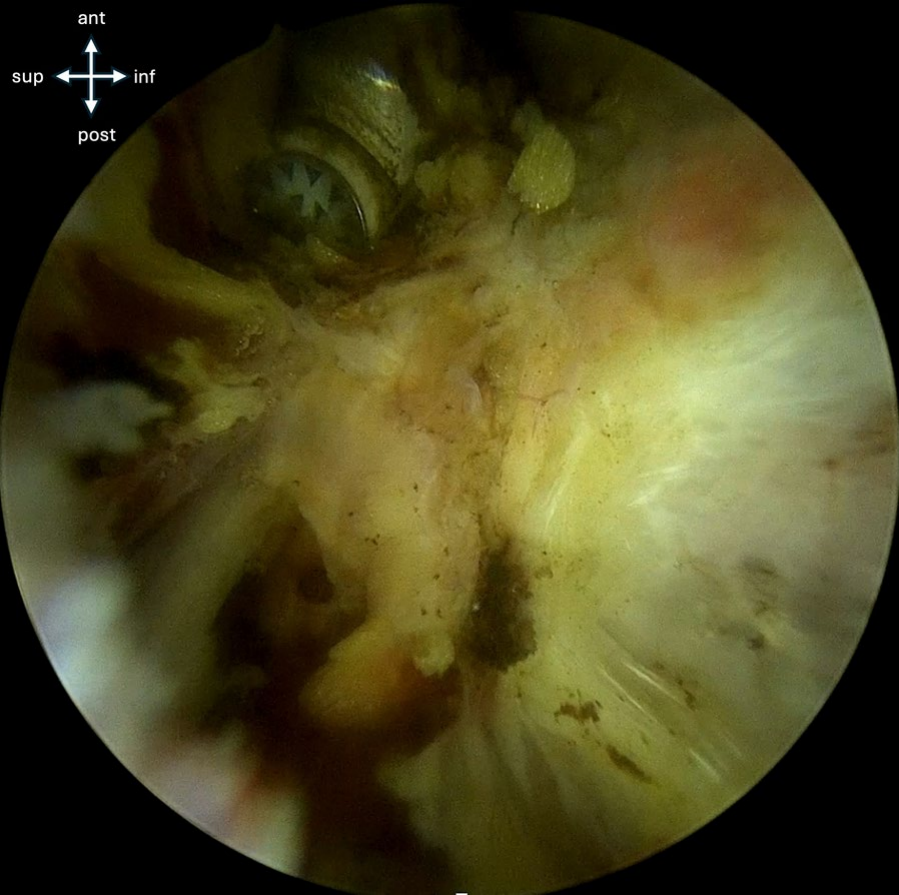
The AIIS avulsed fragment is freed from adherences to the hip capsule and the ilium to facilitate fracture reduction

**Fig. 5:**
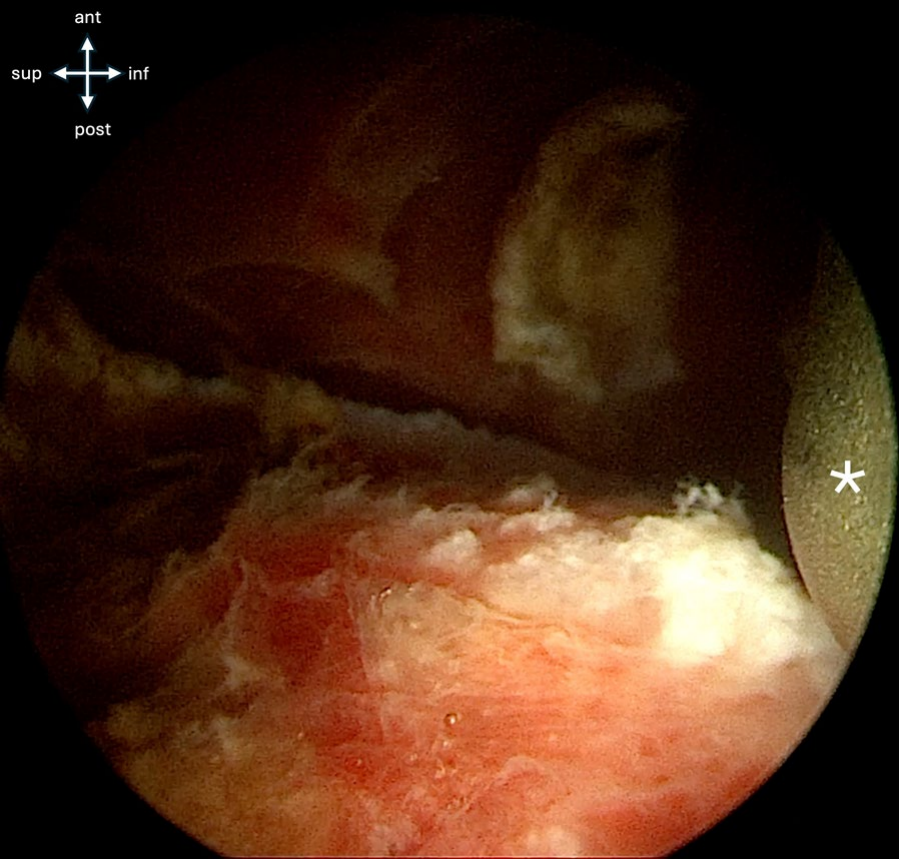
After adherences are cleared, the fracture bed is exposed

Under endoscopic visualization, the fracture site is identified (Fig. 3). A shaver and radiofrequency are used for debridement of the fracture interface (Fig. 4), facilitating mobilization of the fragment (Fig. 5).

**Fig. 6:**
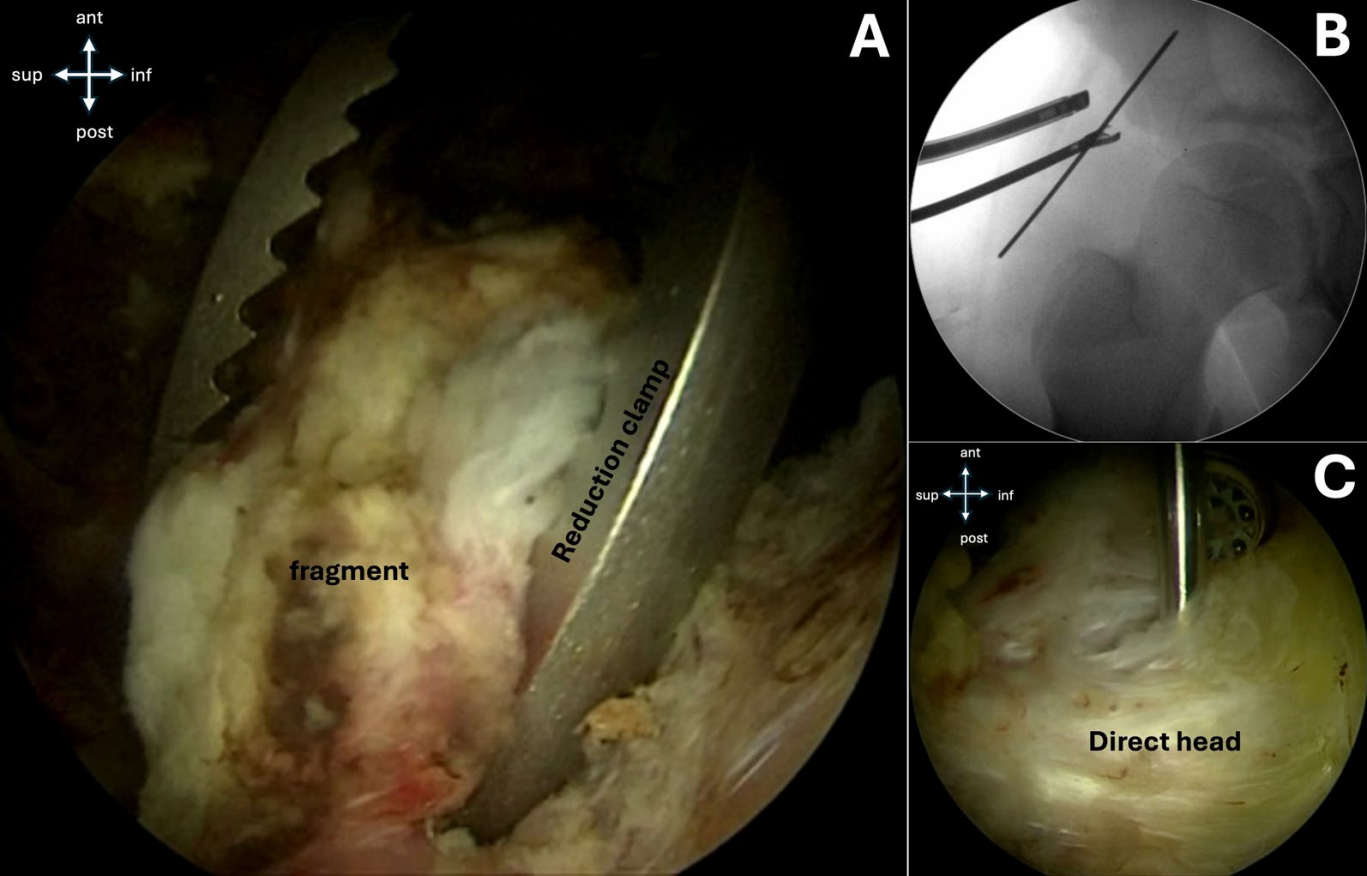
**A** The avulsed fragment is reduced using a reduction clamp. **B** Under fluoroscopic view, fracture reduction is confirmed and a dedicated guide-wire is placed to maintain reduction. **C** Endoscopic view of fracture reduction and proper guide-wire placement

Through the IP, a switching stick or reduction clamp is introduced for the reduction maneuver. The primary objective is anatomical reduction or sufficient proximalization of the avulsed fragment to prevent subspine impingement during hip flexion. If difficulty is encountered in mobilizing the avulsed fragment, hip flexion can be utilized to reduce tension on the rectus femoris muscle. Once reduction is achieved, a 2-mm K-wire is introduced perpendicularly to the fracture site via the IP to provide temporary stabilization. Intraoperative fluoroscopic imaging is used to confirm proper alignment (Fig. 6).

**Fig. 7:**
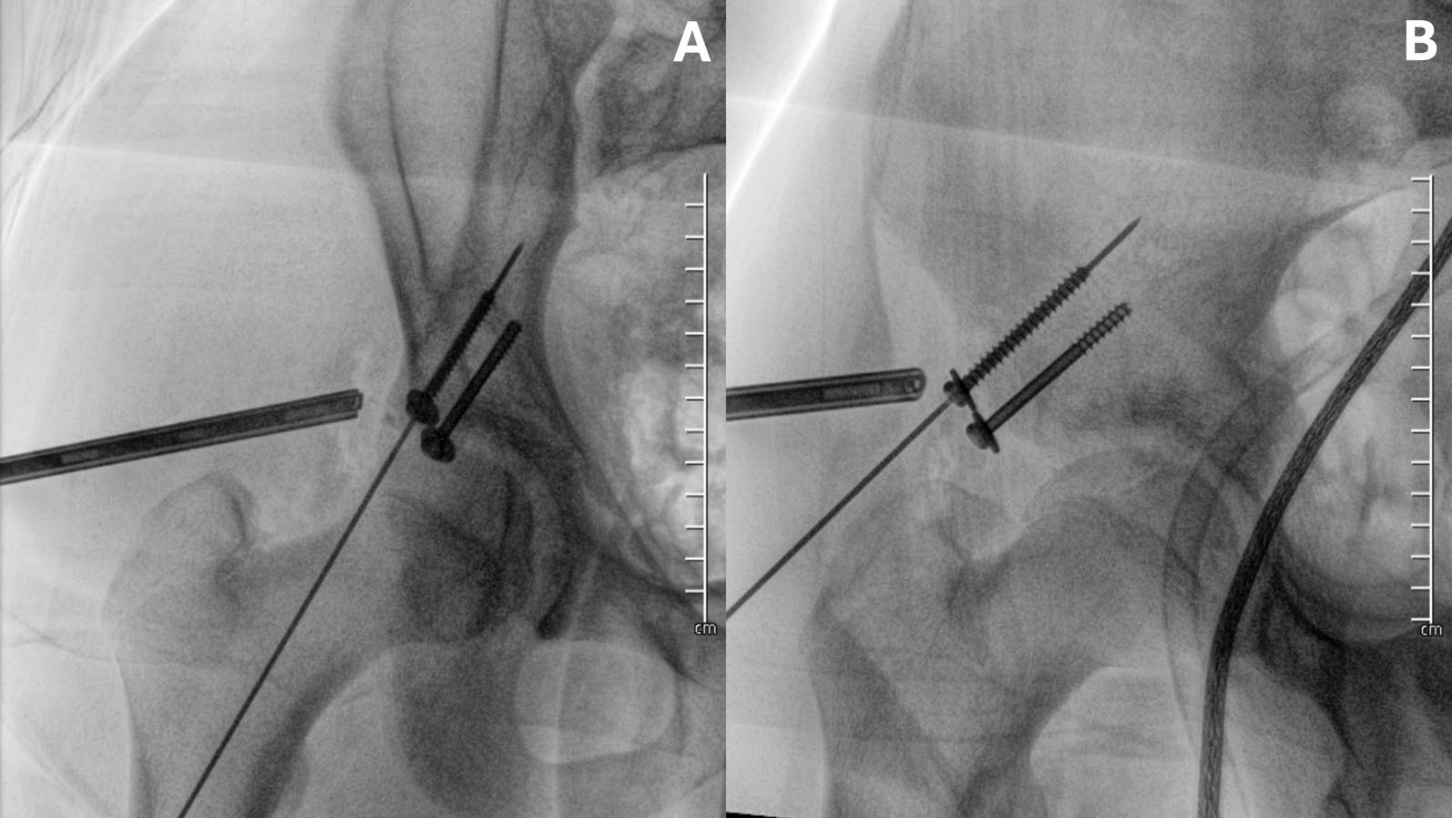
Internal fixation is achieved with two cannulated 4.5-mm screws. Screw placement is checked in AP (**A**) and iliac wing (**B**) pelvis views

Definitive fixation is accomplished with one or two 4.5-mm partially threaded cannulated screw(s) inserted through the IP. The reduction and screw placement are verified using fluoroscopic imaging in Judet’s iliac wing and obturator view (Fig. 7).

**Fig. 8:**
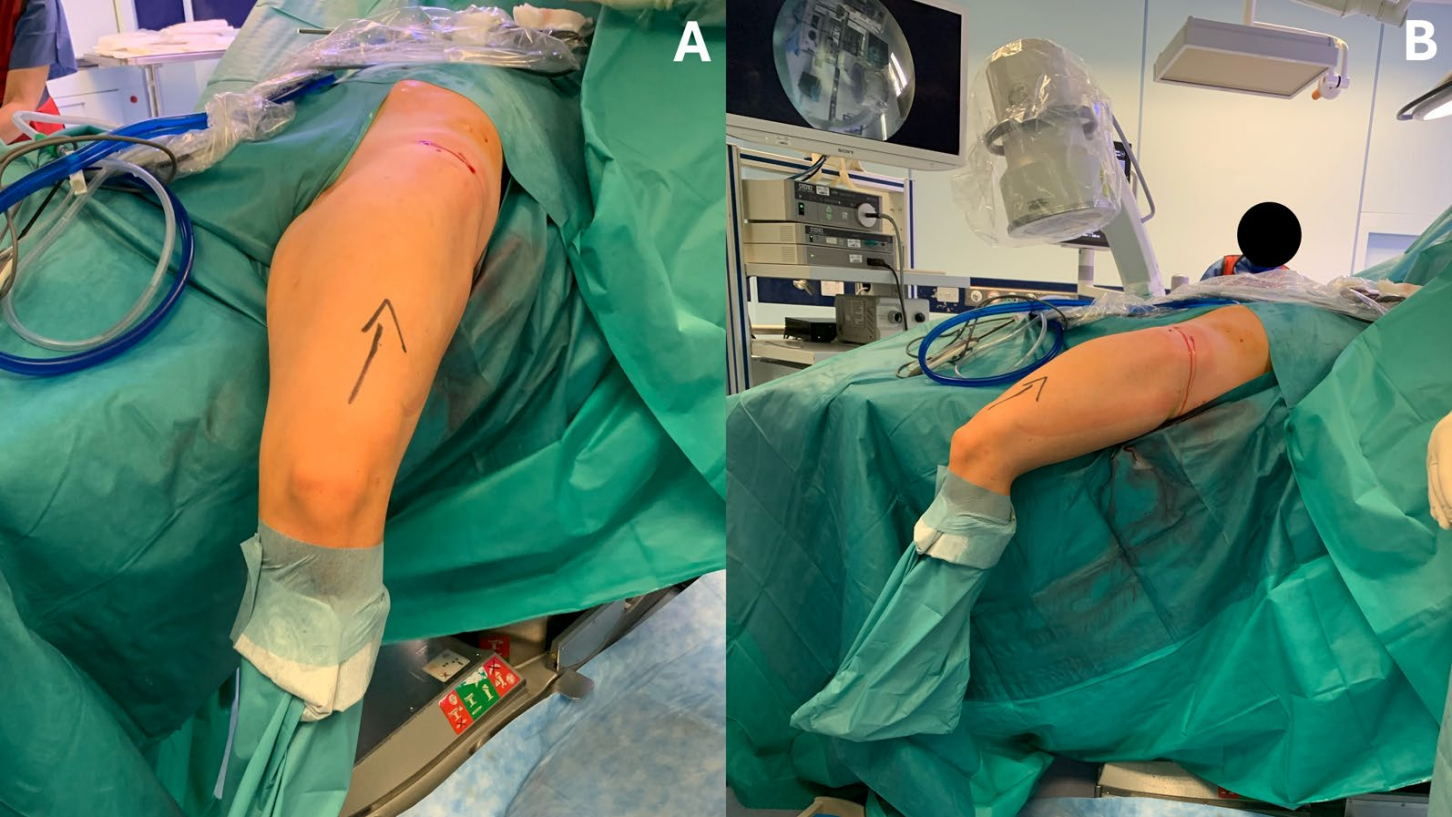
The strength of the internal fixation is assessed intraoperatively by performing hip extension and knee flexion to ensure stability

A stress test, performed through hip extension and knee flexion, is strongly recommended to ensure fixation stability and avoid the risk of secondary displacement (Fig. 8). Finally, the skin is closed with resorbable sutures and a sterile dressing is applied.

**Fig. 9:**
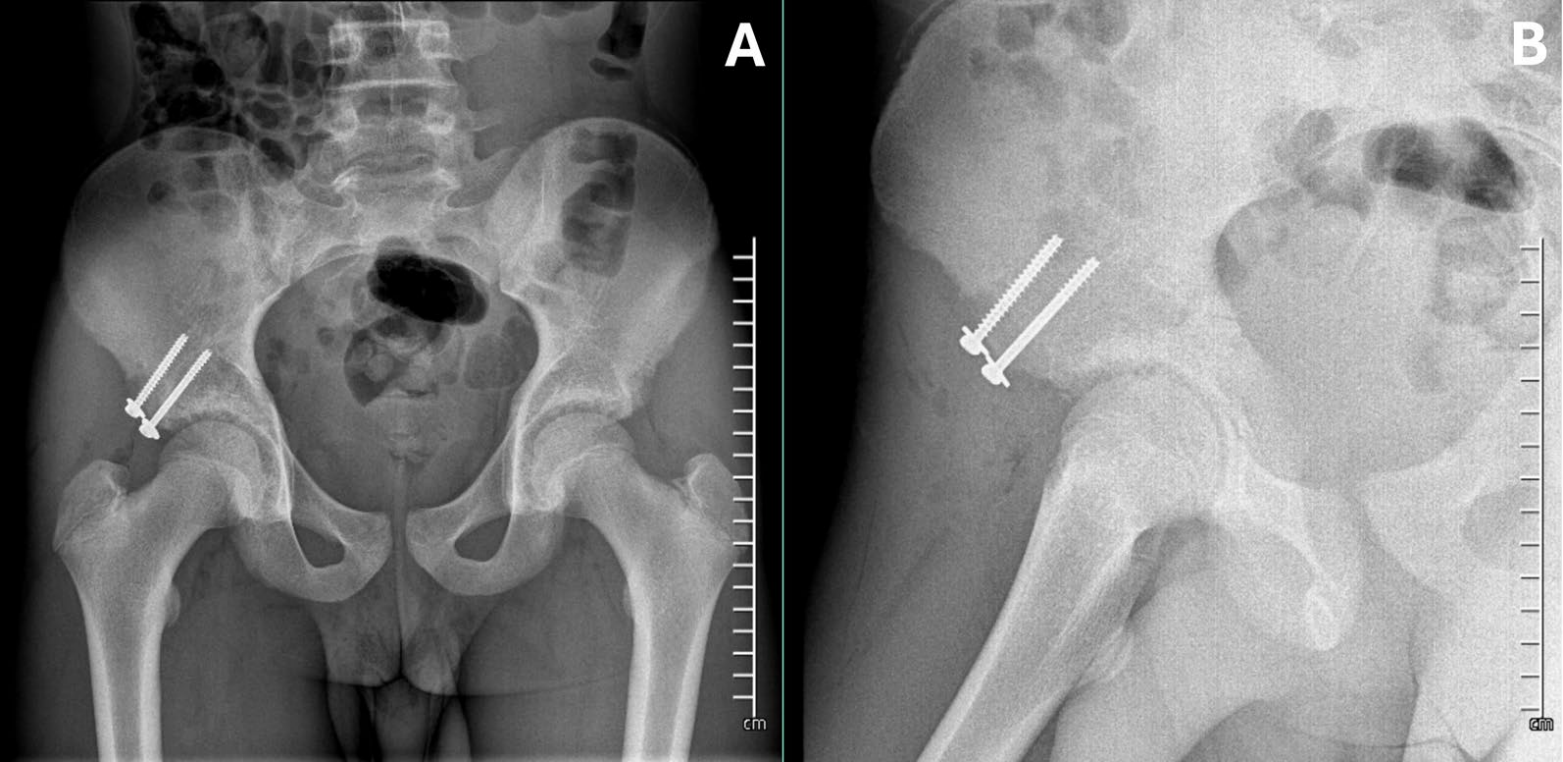
One-year follow-up x-rays in AP pelvis (**A**) and iliac wing (**B**) view showing proper screw placement, fracture healing, and no signs of heterotopic ossification

Patients were restricted to toe-touch weight-bearing with crutches for 4 weeks, then gradually progressing to full weight-bearing. Rehabilitation was structured in two phases. Phase 1 (weeks 1–4) focuses on passive rotational motion exercises to prevent adhesion formation. Phase 2 (weeks 4–12) is the strengthening phase, progressively restoring muscle function. At week 12 postoperatively, the patient was examined before allowing return to sport activities [32]. Figure 9 shows postoperative x-rays at 1-year follow-up.

### Statistical analysis

Categorical data are reported as frequencies and percentages, and continuous variables as mean and standard deviation. A power analysis was conducted to determine adequate sample size to detect a clinically meaningful difference in Modified Harris Hip Score (mHHS) compared with the general population. Given an alpha error of 0.05, a statistical power of 0.80, and a minimal clinically important difference (MCID) of 3% [33], the analysis indicated that a sample size of at least ten patients would be required. The statistical analysis was performed using StataMP13 (Stata Corp).

## Results

Patient demographic and baseline characteristics are reported in Table 1. The study included 11 male patients with mean age of 14.1 years (SD 0.6 years, range: 12.8–15.0 years). The mean time from injury to surgery was 4.2 days (SD 2.6 days, range: 1–11 days). Fractures were classified based on the adapted Hetsroni system [23] as type I in two patients (18.2%), type II in four patients (36.4%), and type III in five patients (45.4%). The mean fracture displacement was 18.3 mm (SD 3.3 mm, range: 15–25 mm). The right side was affected in seven cases (63.6%), while the left side was affected in four cases (36.4%). The follow-up duration was 20.0 months (SD 13.3 months, range: 6–47 months).

**Table 1:** Baseline characteristics

Patient	Age (years)	Sex	Time to surgery (days)	Follow-up (months)	Side	Displacement (mm)	Classification*
1	12.8	M	5	47	R	15	II
2	14.1	M	1	41	R	22	II
3	14.9	M	4	28	L	20	III
4	13.6	M	4	20	R	15	II
5	14.4	M	3	18	R	19	I
6	14.2	M	3	15	L	25	III
7	13.2	M	6	15	R	15	III
8	14.8	M	11	15	L	15	I
9	14.0	M	4	9	R	20	III
10	14.0	M	3	7	R	18	III
11	15.0	M	2	6	L	17	II
Mean ± SD	14.1 ± 0.6		4.2 ± 2.6	20.0 ± 13.3	L: 4/11 (36.4%)	18.3 ± 3.3	Type I: 2/11 (18.2%) Type II: 4/11 (36.4%) Type III: 5/11 (45.4%)

*M* male, *R* right, *L* left, *SD* standard deviation

^*^Classification adapted from Hetsroni et al. for anterior inferior iliac spine (AIIS) acute avulsion fractures

Postoperative outcomes are reported in Table 2. The surgical duration was 71.4 min (SD 17.1 min). The mHHS [28] was 89.7 (SD 3.1), and the UCLA activity score [29, 30] was 9.7 (SD 0.6).

**Table 2:** Postoperative outcomes

	Postoperative
Surgical time (min)	71.4 ± 17.1
MHHS	89.7 ± 3.1
UCLA	9.7 ± 0.6
Fadir test	1/11 (9.1%)
Subspine	1/11 (9.1%)
HO	1/11 (9.1%)
Revision	1/11 (9.1%)
LFCN	1/11 (9.1%)

*M* male, *R* right, *L* left, *mHHS* modified Harris Hip Score, *UCLA* University of California at Los Angeles score, *Subspine* subpsine impingement test, *HO* heterotopic ossification, *Revision* revision surgery, *LFCN* lateral femoral cutaneous nerve neuropathy

One patient (9.1%) experienced secondary displacement after EARIF and required open revision surgery. This patient experienced temporary lateral femoral cutaneous nerve (LFCN) neurapraxia, heterotopic ossification (HO, Brooker grade 1), symptoms of subspine impingement, and positive FADIR sign.

Radiographic evaluations confirmed fracture healing in all cases without evidence of malunion or nonunion. All patients returned successfully to sports activities within 6 months following surgery.

## Discussion

The management of anterior inferior iliac spine (AIIS) avulsion fractures, particularly those with significant displacement, remains controversial [1]. Our study presents evidence supporting the use of endoscopic-assisted reduction and internal fixation (EARIF) for treating displaced AIIS avulsion fractures, offering a minimally invasive alternative to traditional open procedures [8–13, 34–38].

Most of the literature relies on the historical threshold of 2 cm of displacement to guide surgical indications for pelvic avulsion fractures [27]. However, this value lacks robust scientific validation and is often applied indiscriminately across different apophyseal injury locations.

The need for a classification system that accounts for the future risk of subspine impingement in displaced anterior inferior iliac spine (AIIS) avulsion fractures stems from the growing recognition of its clinical implications. Subspine impingement, a subtype of femoroacetabular impingement (FAI), results from abnormal contact between the femoral head_neck junction and an excessively prominent or malunited AIIS. This condition has been well documented to cause hip pain, functional limitations, and early-onset osteoarthritis if left untreated. Hetsroni et al. [22] demonstrated the mechanical role of AIIS morphology in hip impingement and proposed a classification system based on the relationship of the AIIS to the acetabular rim, highlighting how certain morphologies (type II and type III) predispose patients to hip dysfunction during flexion and internal rotation.

Adapting Hetsroni’s classification to acute AIIS fractures offers a framework for understanding which fracture patterns pose the highest risk of subspine impingement if malunion occurs. In our study, we categorized fractures into three types based on the fragment’s position relative to the acetabular rim: type I (proximal to the rim), type II (aligned with the rim), and type III (distal to the rim). This adapted classification may assist in stratifying fracture severity on the basis of its potential risk of subspine impingement. However, long-term follow-up studies are necessary to validate its reliability and clinical relevance. Ensuring proper reduction and fixation of type II and III fractures may play a crucial role in minimizing the risk of chronic impingement-related symptoms.

The clinical importance of this approach is underscored by the growing body of evidence demonstrating the efficacy of arthroscopic decompression for managing chronic subspine impingement [14, 20–22]. By addressing the risk factors for impingement during the acute phase of AIIS fracture management, our classification system aims to prevent the long-term complications of malunion, which are often challenging to treat and can significantly impair athletic performance and quality of life in young, active patients.

Our findings report satisfactory radiographic and clinical outcomes for the EARIF technique [25] in patients with displaced AIIS fractures. At a mean follow-up of 20 months, patients achieved excellent functional outcomes, as evidenced by high modified Harris Hip Score (mHHS) and UCLA activity scores. Radiographic evaluation confirmed fracture healing in all cases, with no instances of malunion or nonunion.

Notably, one patient required open revision surgery due to secondary displacement after EARIF. This underscores the importance of achieving a stable fixation. This complication occurred in one of the first cases. Since then, the fixation’s stability is routinely checked intraoperatively with hip extension and knee flexion under fluoroscopic view.

Open surgery through an anterior approach has been associated with higher risk of heterotopic ossification and LFCN neuropathy [1, 39]. On the other hand, conservative treatment was associated with painful nonunion and hypertrofic malunion leading to subspine impingement [4, 18, 40–43]. No such complications were reported in the other cases treated with EARIF and that did not require revision surgery. EARIF could potentially minimize complications associated with an open surgical dissection and the risks of later complications. Further studies are warranted to confirm such hypothesis.

This study presents several limitations. Firstly, the study’s small sample size may limit the generalizability of the findings, and larger cohorts are needed to confirm the results. Secondly, the relatively short follow-up does not allow for long-term outcome assessment, particularly regarding the risk of subspine impingement and joint degeneration. Thirdly, the study lacks a control group, preventing direct comparisons with nonoperative management outcomes. Furthermore, the adapted Hetsroni classification for AIIS avulsion fractures is not yet validated, and no other standardized classifications exist to guide surgical treatment, which may impact reproducibility and clinical decision-making. Finally, the study was conducted in a single, high-volume center, which may limit its applicability to different clinical settings. Future research with multicenter trials and longer follow-up periods is recommended to strengthen these findings and validate the proposed classification system.

## Conclusions

EARIF provides an effective and minimally invasive solution for treating displaced AIIS avulsion fractures. The introduction of a systematic classification approach may help guide treatment decisions and optimize acute fracture management. However, further long-term studies are needed to evaluate its impact on reducing the risk of subspine impingement and other chronic complications.

## Data Availability

A paper copy of the database is available at Città della salute e della Scienza di Torino.
